# Potential Immunogenic Activity of Computationally Designed mRNA- and Peptide-Based Prophylactic Vaccines against MERS, SARS-CoV, and SARS-CoV-2: A Reverse Vaccinology Approach

**DOI:** 10.3390/molecules27072375

**Published:** 2022-04-06

**Authors:** Taimoor Khan, Abbas Khan, Jawad Khaliq Ansari, Muzammil Hasan Najmi, Dong-Qing Wei, Khalid Muhammad, Yasir Waheed

**Affiliations:** 1 Department of Bioinformatics and Biological Statistics, School of Life Sciences and Biotechnology, Shanghai Jiao Tong University, Shanghai 200240, China; taimor.khaan@outlook.com (T.K.); abbaskhan@sjtu.edu.cn (A.K.); dqwei@sjtu.edu.cn (D.-Q.W.); 2 Foundation University Medical College, Foundation University Islamabad, Islamabad 46000, Pakistan; dr.jkansari@gmail.com (J.K.A.); najmimh@hotmail.com (M.H.N.); 3 Peng Cheng Laboratory, Vanke Cloud City Phase I Building 8, Xili Street, Nashan District, Shenzhen 518055, China; 4 State Key Laboratory of Microbial Metabolism, Shanghai-Islamabad-Belgrade Joint Innovation Center on Antibacterial Resistances, Joint Laboratory of International Cooperation in Metabolic and Developmental Sciences, Ministry of Education and School of Life Sciences and Biotechnology, Shanghai Jiao Tong University, Shanghai 200240, China; 5 Department of Biology, College of Sciences, United Arab Emirates University, Al Ain 15551, United Arab Emirates

**Keywords:** MERS-CoV, SARS-CoV, SARS-CoV-2, prophylactic vaccine, epitopes

## Abstract

The continued emergence of human coronaviruses (hCoVs) in the last few decades has posed an alarming situation and requires advanced cross-protective strategies against these pandemic viruses. Among these, Middle East Respiratory Syndrome coronavirus (MERS-CoV), Severe Acute Respiratory Syndrome coronavirus (SARS-CoV), and Severe Acute Respiratory Syndrome coronavirus-2 (SARS-CoV-2) have been highly associated with lethality in humans. Despite the challenges posed by these viruses, it is imperative to develop effective antiviral therapeutics and vaccines for these human-infecting viruses. The proteomic similarity between the receptor-binding domains (RBDs) among the three viral species offers a potential target for advanced cross-protective vaccine designs. In this study, putative immunogenic epitopes including Cytotoxic T Lymphocytes (CTLs), Helper T Lymphocytes (HTLs), and Beta-cells (B-cells) were predicted for each RBD-containing region of the three highly pathogenic hCoVs. This was followed by the structural organization of peptide- and mRNA-based prophylactic vaccine designs. The validated 3D structures of these epitope-based vaccine designs were subjected to molecular docking with human TLR4. Furthermore, the CTL and HTL epitopes were processed for binding with respective human Lymphocytes Antigens (HLAs). In silico cloning designs were obtained for the prophylactic vaccine designs and may be useful in further experimental designs. Additionally, the epitope-based vaccine designs were evaluated for immunogenic activity through immune simulation. Further studies may clarify the safety and efficacy of these prophylactic vaccine designs through experimental testing against these human-pathogenic coronaviruses.

## 1. Introduction

Human coronaviruses (hCoVs) form a diverse group of single-stranded RNA viruses [1]. Among the four genera of CoVs (alpha, beta, gamma, and delta), α-coronavirus and β-coronavirus are highly pathogenic to humans [2]. This is reflected by the severe disease outcomes caused in humans due to highly pathogenic hCoVs that mainly involve MERS-CoV, SARS-CoV, and SARS-CoV-2 [3,4]. These three spherical-shaped hCoVs affect human lungs by targeting the lower respiratory tract and may ultimately lead to organ dysfunction and fatality [5]. Previously, the emergence and transmission of MERS-CoV and SARS-CoV caused catastrophic effects around the globe [6,7]. Despite the associated threats of these pandemic-causing hCoVs, the recent global outbreak of SARS-CoV-2 further aggravated the situation [8]. These human-infecting CoVs have a genome size spanning from 26 to 32 kb [9,10]. This includes MERS-CoV, which has a genome size of approximately 30 kb, while SARS-CoV and SARS-CoV-2 have a smaller size of approximately 29 kb [11]. The genomic similarity of the recently emerged SARS-CoV-2 with SARS-CoV and MERS-CoV is approximately 79.5% and 50%, respectively [12]. Moreover, these hCoVs have a typical genomic organization that consists of 5′-methylated caps and 3′-polyadenylated tails [8,13]. The 3′-terminal genomic region is mainly responsible for encoding the viral life cycle regulatory proteins (spike protein, nucleocapsid protein, membrane protein, and envelope protein). On the other hand, the 5′-terminal genomic region encodes for the nonstructural proteins that are involved in viral replication [13,14]. The structural and functional profiling of related proteins of hCoVs provides a strong basis to understand viral pathogenesis. On the basis of this knowledge, advanced cross-protective strategies may be designed to combat these human-pathogenic CoVs.

The proteomic organization of hCoVs includes the spike protein, which has an important structural and functional role. The spike protein mainly plays a key role in mediating the CoV’s interaction with the host cell [15]. Spike proteins contain two main subunits called S1 and S2. The receptor-binding domain (RBD) in the S1 subunit mainly regulates the binding of hCoVs with host receptors. Moreover, the S2 subunit mediates the fusion of hCoVs with host membranes and regulates the penetration of the viral genome into the cytoplasm of the host cells [16]. The host cell entry of the three highly pathogenic hCoVs is mainly mediated by the binding of the RBD to the host’s cell surface functional receptors [17]. Despite the important function of binding receptors, spike proteins also regulate the process of hCoVs’ host cell entry [18]. Additionally, for spike protein priming, these viruses deploy the cellular serine protease TMPRSS2 and the endosomal cysteine proteases cathepsin B and L to mediate the entry of the virus into host cells [17,19].

The proteomic similarity of the three highly pathogenic hCoVs may be utilized to set up precise strategies to develop cross-protective therapeutics against these deadly pathogens [20,21,22]. Although remarkable advances have been made in therapeutics development against lethal hCoVs [23,24,25], advanced efforts are needed to develop prophylactic vaccines against them. Previously, there were no available cross-protective vaccines against pandemic-causing hCoVs. Herein, immunoinformatics-based approaches were adapted to design a prophylactic vaccine against three highly pathogenic hCoVs. In this work, the RBD-containing region of the three species was targeted for potential vaccine designs (peptides and mRNA). This was achieved through domain-specific potential epitope screening followed by the structural design of two different prophylactic vaccine designs. Herein, several linkers (EAAK, AAY, GPGPG, and KK) were used to join the adjuvant and different immune epitopes together [24]. The peptide-based prophylactic vaccine design was evaluated for potential immunogenic activity through molecular docking, in silico cloning, and immune simulation approaches. Additionally, the predicted epitopes were evaluated through molecular docking to explore the interaction patterns of different epitopes with HLA (Human Leukocyte Antigen) molecules. Our study provided in silico, strategy-based prophylactic vaccine designs against three hCoVs. The aim of the study was to design a prophylactic vaccine that included epitopes from the different SARS species. Due to the cross-protective properties of the designed vaccine, it is suitable for being used as an advanced therapeutic against any of these viruses. Moreover, such a vaccine design provides protection from coinfections of SARS from different strains and may be able to produce immunity against a wider range of viral species. Further studies with experimental validations may clarify the clinical manifestations associated with the vaccine designs against these highly pathogenic human coronaviruses.

## 2. Results

### 2.1. Sequences Retrieval

The spike glycoprotein containing the RBD sequences of MERS-CoV, SARS-CoV, and SARS-CoV-2 was downloaded from UniProt. The accession details of each domain of the three different coronaviruses that were evaluated for further screening for putative T-cell, B-cell, HTL, and IFN epitopes are given in Table 1.

### 2.2. Prioritization of Putative Vaccine Epitopes

Different epitopes recognized by the immune system were characterized as a prerequisite for peptide-based vaccine designs. Herein, each RBD protein sequence of MERS-CoV, SARS-CoV, and SARS CoV-2 was subjected to putative vaccine epitope screening. This was followed by the selection of T-cell, HTL, and B-cell epitopes for the final prophylactic vaccine designs.

#### 2.2.1. T-Cell Epitopes

An immune response mainly requires T-cell-specific recognition of antigenic epitopes for robust induction. These T-cell-recognized epitopes are vital to combat invading pathogens from developing infections [26]. Moreover, the potential of T-cell epitope prediction based on binding specificity through in silico approaches offers wide utilities [27]. T-cell epitopes were predicted for each RBD sequence of the three different coronaviruses. The total number of identified T-cell epitopes for MERS-RBD was 11. In addition, nine epitopes were predicted for SARS-RBD and for SARS-CoV-2-RBD. All the identified T-cell epitopes were further screened to select putative vaccine epitopes. After evaluating the potential antigenicity and allergenicity status of each predicted T-cell epitope, only the one with high immunogenic potential (Table 2) was included in further vaccine-designing procedures.

#### 2.2.2. HTL Epitopes

HTL epitopes play a pivotal role in the induction of a protective immune response against invading pathogens [28]. They are involved in B-cell activation and maturation. The inclusion of HTL epitopes in peptide-based vaccine constructs helps in the development of safe and effective therapies against human pathogens [29]. Herein, the HTL epitope screening for each RBD of the different CoV species was performed. The numbers of identified HTL epitopes for MERS, SARS-CoV, and SARS-CoV-2 were 1351, 1456, and 1463, respectively. All the identified HTL epitopes were further screened to select putative vaccine epitopes. After mapping HTL epitopes on the basis of antigenic potential, selected epitopes (Table 3) were included in further vaccine design procedures.

#### 2.2.3. B-Cell Epitopes

B-cell epitope mapping plays a key role in robust immune response induction. This is due to the important role of B-cells in the regulation of an immune response through the production of antibodies against the invading pathogen [30,31]. This B-cell response is triggered by the identification of epitope sequences by the antigen. Moreover, the identification of highly immunogenic B-cell epitopes is a prerequisite in peptide-based vaccine designs [32,33,34]. The total number of B-cell epitopes was predicted for each CoV species. The numbers of identified B-cell epitopes for MERS, SARS-CoV, and SARS-CoV-2 were 18, 24, and 23, respectively. All the identified B-cell epitopes were further screened to select putative vaccine epitopes. After mapping, selected T-cell epitopes with high immunogenic potential (Table 4) were included in further vaccine design procedures.

### 2.3. Epitopes–HLAs Molecular Docking

Peptides are presented to T-cells by HLA molecules, which results in the T-cell immune response. HLA molecules are highly polymorphic in nature; hence, peptides capable of interacting with HLA molecules are potent vaccine candidates [35]. In silico molecular docking was carried out using the HawkDock server to analyze the binding behavior of the CTL and HTL epitopes with their respective HLAs. The list of species-specific CTLs and HTLs with their respective HLAs is provided in Table 5. The 3D models of nine HLAs were retrieved from the protein databank, which included HLA-B*57:01 (PDB ID: 3X11), HLA-B*35:01 (PDB ID: 4PRA), HLA-A*01:01 (PDB ID: 6MTM), HLA-B*58:01 (5VWJ), HLA-B*15:01 (6UZP), HLA-A*24:02 (7MJA), HLA-A*02:06 (3OXR), HLA-B*51:01 (4MJI), and HLA-A*68:01 (6PBH), and prepared for docking. Higher binding energies obtained after the docking analysis of the studied CTL and HTL epitopes with their corresponding HLAs indicated their potential ability to be utilized in the vaccine design. The details, including peptide sequences, corresponding HLAs, and binding energies for each of the docking complexes, are given in Table 5. We characterized CTLs as MHC Class I (MHC-I) and HTLs as MHC Class II (MHC-II) binding epitopes with their corresponding HLAs.

**Figure 1:**
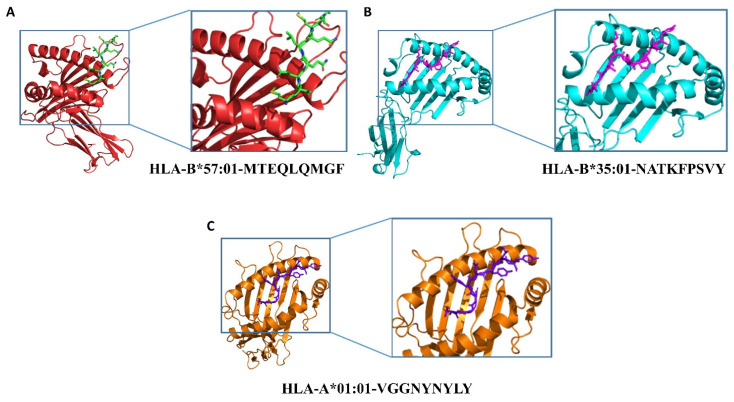
Docking complexes of selected CTL epitopes for each CoV species with respective HLAs. Whereas (**A**–**C**) shows the different-colored (red, cyan, and orange) complexes, green, purple, and marine blue represent each class of HLA molecule and the small peptide (epitope) used for docking.

For the docking complexes of the CTL epitopes with their corresponding HLAs, the total binding free energies were −27.86 kcal/mol for HLA-B*57:01-MTEQLQMGF, −20.65 kcal/mol for HLA-B*35:01-NATKFPSVY, and −21.87 kcal/mol for HLA-A*01:01-VGGNYNYLY. The CTL–HLA docking complexes are shown in Figure 1; the binding free energy calculations and interaction patterns are given in Table S1 (see Supplementary Material). For the docking complexes of the HTL epitopes with their corresponding HLAs, the total binding free energies were −37.1 kcal/mol for HLA-B*58:01-LSMKSDLSVSSAGPI, −31.49 kcal/mol for HLA-B*15:01-QFNYKQSFSNPTCLI, −33.44 kcal/mol for HLA-A*24:02-NYNYKYRYLRHGKLR, −35.7 kcal/mol for HLA-A*02:06-SGDVVRFPNITNLCP, −29.69 kcal/mol for HLA-B*51:01-TESIVRFPNITNLCP, and −46.27 kcal/mol for HLA-A*68:01-RFASVYAWNRKRISN. The HTL–HLA docking complexes are shown in Figure S1 (see Supplementary Material); the binding patterns of HTL–HLAs are given in Figure S2 (see Supplementary Material), and the binding free energy calculations are given in Table S2 (see Supplementary Material).

### 2.4. Prophylactic MEVC Designs

The use of computational vaccinology approaches accelerates the design of protective vaccines with increased safety and efficacy. Advanced trends including the development of predisposed biological resources have been adapted to accelerate vaccine designing against human-pathogenic species [22,23,24]. The identification of highly specific vaccine constructs on the basis of the inclusion of immunogenic epitopes [21] is vital for the induction of robust immune responses against pathogens. These vaccine constructs are required to be highly immunogenic and nonallergenic. Moreover, the use of proper adjuvants and linkers is required for the proper designing of the final vaccine construct. After screening, all the putative epitopes were subjected to the construction of multiepitope-based and mRNA-based constructs. Two different prophylactic vaccines named MEVC-mRNA-CoV and MEVC-CoV were designed against the three target CoV species. This was achieved through joining the different putative epitopes (Table 2, Table 3 and Table 4) with the help of different linkers, i.e., EAAK, AAY, GPGPG, and KK, for epitope-based vaccine designs. The use of these linkers increases structural stability by avoiding self-folding and enhances the immunogenicity of protein sequences [36]. All the constructed vaccine sequences were further evaluated for immunogenicity potential. This was performed through the analysis of each vaccine sequence on the basis of antigenicity and allergenicity status. The details of all the designed multiepitope-based vaccine constructs along with immunogenicity status are given in Table 5.

#### 2.4.1. mRNA-Based Vaccine Construction

**Figure 2:**
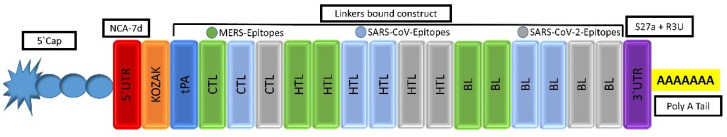
The final mRNA vaccine construct with organized order of elements from left (5′) to right (3′). Each structural element including 5′ cap, 5′ UTR, Kozak sequence, signal protein, CTL epitopes, HTL epitopes, B-cell epitopes, 3′ UTR, and a poly (A) tail are represented by different color scheme.

An mRNA-based vaccine was designed by targeting the RBD of the selected three hCoV species. This comprised joining together the epitopes predicted for the RBDs of all the studied species in the final vaccine construct (MEVC-mRNA-CoV). The final vaccine construct contained four CTL epitopes, four HTL epitopes, and six linear B-cell epitopes. The selected CTL, HTL, and linear B-cell epitopes were placed together using AAY, PMGLP, and GGGGS linkers, respectively. The 5′ m7G cap was placed right before the NCA-7d (5′ UTR) followed by the Kozak sequence and signal peptide (tissue Plasminogen Activator). The mRNA vaccine sequence spanned from the tPA to the last LBL epitope, where a stop codon was added right before the S27a+R3U (3′ UTR) and poly (A) tail (120 nucleotides long). The selected CTL, HTL, and B-cell epitopes are provided in Table 2, Table 3 and Table 4, respectively. The arrangements of the mRNA vaccine components are graphically illustrated in Figure 2.

#### 2.4.2. Multiepitope-Based Vaccine Construct

**Figure 3:**
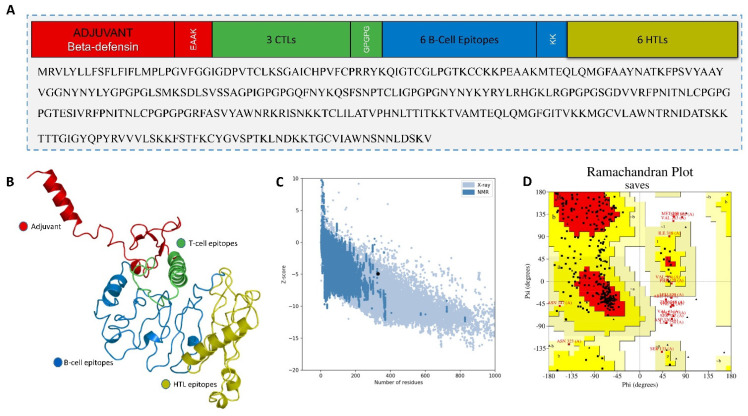
Topological organization, 3D structures, and validation of the MEVC-CoV. (**A**) represents the topological organization of the vaccine design with subunits and linkers colored differently; (**B**) shows the 3D modeled structures of the vaccine construct; and (**C**,**D**) show the ProSA-Web and Ramachandran plot validation, respectively.

The final vaccine construct containing the putative CTL, HTL, and B-cell epitopes was joined using different linkers, as shown in Figure 3A. Next, the linear vaccine constructs were subjected to 3D modeling for the depiction of the MEVC structures. By utilizing the structural modeling package Robetta, all the MEVC structures were modeled on the basis of the amino acid sequences. This was performed by submitting these sequences into the online server using the Robetta Fold option, and the obtained structures were refined using PyMOL. The different 3D models generated for each MEVC are given in Figure 3B. Moreover, all the modeled structures for each MEVC were validated through Ramachandran and ProSA-Web analyses. These analyses revealed a Z score of −5.24 (Figure 3C) and a large number (>80%) of residues in the favored region (Figure 3D), reflecting high confidence for the structure. Moreover, the calculated ERRAT quality factor was >85, and approximately 80 percent of the residues averaged a 3D–1D score ≥0.2. All these positive scores indicated a stable nature and validated the vaccine structure.

The total length of MEVC-Prophylactic-CoV was 329 amino acids, and it had a high antigenicity score of 0.73 and showed a nonallergenic nature. The final prophylactic vaccine was evaluated for numerous physiochemical properties to ensure the safety and efficacy of the vaccine design. During this process, JCat (http://www.jcat.de, accessed on 26 November 2021) was utilized; after providing the input protein sequence, we selected E. coli strain-K12 as the target organism. This was followed by further analyses and choosing the “Avoid rho-independent transcription terminators” and “Avoid prokaryotic ribosome binding sites” options in the online server. The GC content (51.67%), CAI value (0.96), molecular weight (35kd), theoretical pI (9.82), and several other analyzed parameters of MEVC-Prophylactic-CoV are shown in Table S3 (see Supplementary Material). These results reflected the hydrophilic and stable nature of the designed MEVC.

### 2.5. In Silico Cloning of the MEVC

**Figure 4:**
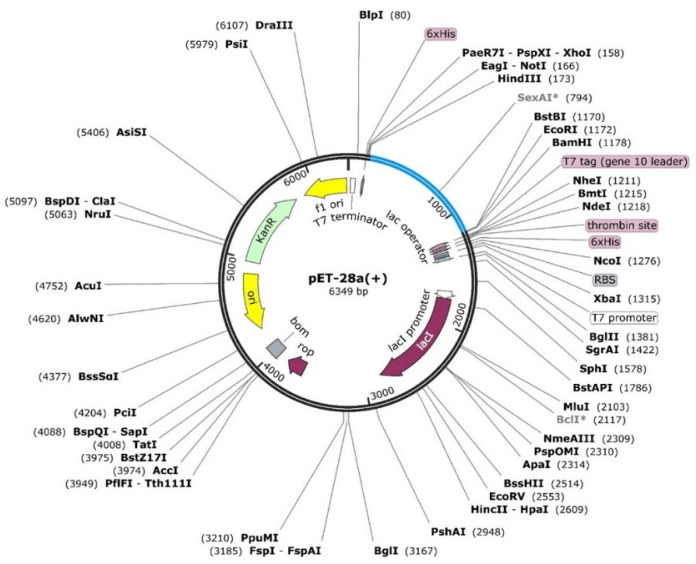
Constructed plasmid map Pet28a+ with blue-colored insert for the designed MEVC.

Cloning is vital to acquire the optimized DNA sequence and insert it properly in the E. coli expression vector before proceeding into further experimental procedures. Using in silico modeling approaches, we designed the corresponding clone for the MEVC-CoV construct. This was achieved through codon optimization, followed by confirmation of putative expression in the E. coli (K-12) strain as represented by the CAI value of the construct. The optimized sequence was added to a Pet28a + vector with the utility of SnapGene software (from Insightful Science; available at snapgene.com). The specific sites of restriction enzymes selected for insertion of the optimized sequence into the vector were EcoR1 and Xho1. The final plasmid map obtained for MEVC-CoV is shown in Figure 4.

### 2.6. Immune Simulation of the MEVC

**Figure 5:**
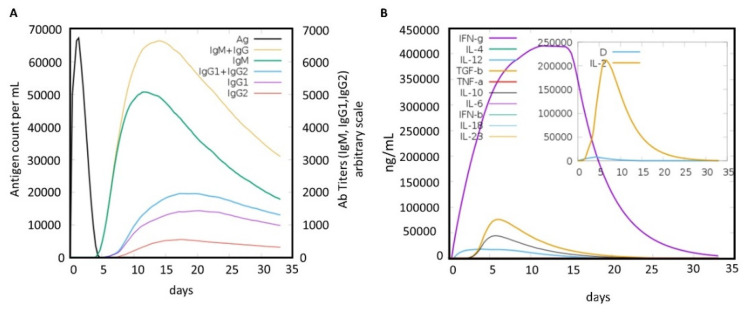
A graphical image of the immune response against the studied pathogenic viruses. Figure (**A**) represents the antibody titers produced, while (**B**) represents cytokine and interleukin production upon the assessment of immune response induction potential of the designed prophylactic-MEVC-CoV.

Using an immune simulation approach, the potential immune response induction with predicted antibody titers produced against the MEVC-prophylactic-CoV construct was evaluated. As was predicted (Figure 5), the injected antigen, after achieving the highest antigen count at day 2, was slowly neutralized until day 5. Afterwards, a strong antibody (IgM and IgG) response was observed, and the highest achieved titer of >6000 counts/mL (arbitrary scale) occurred between 10 and 15 days. A high of approximately >6000 counts/mL for combined IgM and IgG, followed by an IgM-specific antibody titer of approximately 5000 counts/mL, was obtained for the MEVC. However, further demonstrations may clarify the depicted higher immunogenic potential of the designed MEVC and the development of protective immunity against hCoVs. The final immune simulation graph obtained for the prophylactic MEVC is shown in Figure 5. Moreover, the prophylactic vaccine design was checked for the potential induction of responses by interleukins (ILs), interferon (IFN), transforming growth factors (TGFs), and transforming necrosis factor (TNF). Among these factors, the highest response produced against the antigenic vaccine was by IFN-γ with a maximum peak (>400,000) observed between days 10 and 15.

### 2.7. Molecular Docking of MEVC with Human TLR4

**Figure 6:**
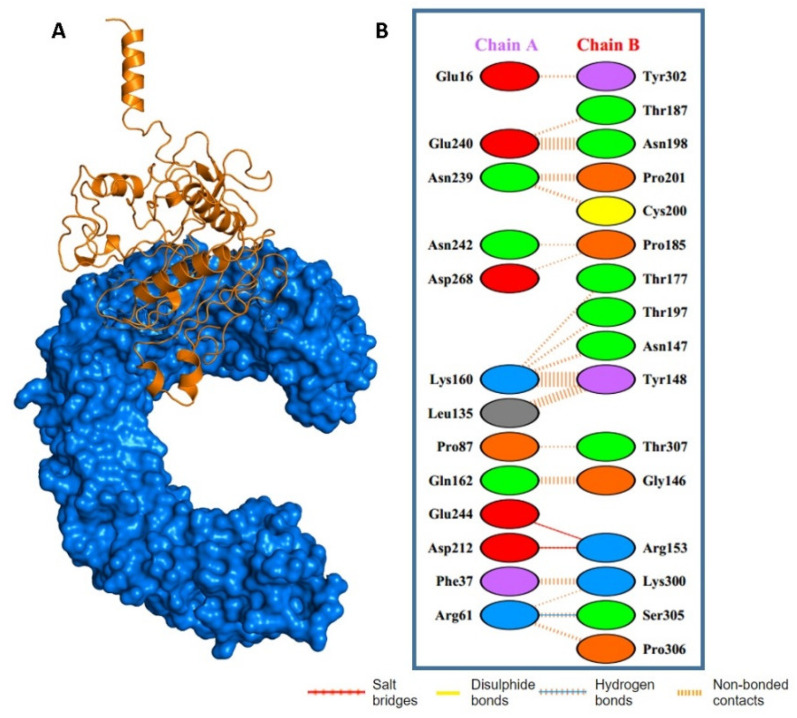
Docking complex and interaction patterns of MEVC-TLR4. Figure (**A**) represents the docking complex for prophylactic-MEVC-CoV. Figure (**B**) represents the binding interactions of MEVC with human TLR4.

The potential interactions of the designed prophylactic vaccine with human immune receptor TLR4 through molecular docking analysis were also explored. Toll-like receptors (TLRs) play a crucial role in the activation of innate immunity by detecting conserved pathogen-associated molecular patterns (PAMPs) and the production of an adaptive immune response against the invading pathogens. Specifically, TLR4 was implicated in the recognition of hCoV structural proteins related to immune response induction. The docking complexes for each of the designed prophylactic MEVCs and TLR4 are shown in Figure 6. A docking score of −42.36 kcal/mol was observed for the MEVC–TLR4 complex. Furthermore, the PDB sum [25] was used to determine the interaction patterns between the prophylactic MEVC and human TLR4. Additionally, one hydrogen bond and two salt bridges were found in the MEVC-CoV + TLR4 docking complex. The details of the binding scores and interaction patterns, including the number of hydrogen bonds and salt bridges for the docking complexes, are given in Figure 6.

## 3. Materials and Methods

### 3.1. RBD Sequence Retrieval

The amino acid sequences of the receptor-binding domains of the three target hCoVs (MERS, SARS-CoV, and SARS-CoV-2) were downloaded from the Universal Protein Knowledge Base (https://www.uniprot.org/, accessed on 2 June 2021) [37].

### 3.2. Putative Vaccine Epitope Screening

Putative epitopes specific to RBD sequence of each studied species were characterized on the basis of the utility of various online available servers. This was followed by immunogenic potential (antigenicity and allergenicity nature) evaluation of the individually characterized immune epitopes (CTL, HTL, B-cell) for each RBD of the studied hCoV species.

#### 3.2.1. Prediction of CTL Epitopes

The prediction of CTL epitopes for each RBD of the three hCoV species was carried out with NetCTL 1.2 server (http://www.cbs.dtu.dk/services/NetCTL/, accessed on 3 June 2021) [38] at default threshold of 0.75. Epitope selection was performed on the basis of the obtained descending order of the combined score. The server predicts MHC-I receptor-specific binding affinity of the input epitope sequence. It utilizes an artificial neural network (ANN) approach to quantify the binding affinity of epitope toward MHC-I. The server also offers wide sensitivity and follows a standard calculation method based on 216 experimentally identified epitopes covering all 12 confirmed HLA supertypes [38].

#### 3.2.2. Prediction of HTL Epitopes

The predictions of HTL epitopes for each RBD of the selected viral species were conducted using IEDB MHC-II module (http://www.iedb.org/, accessed on 8 June 2021) [39], corresponding to a reference set of seven HLAs. Herein, default parameters were used to allow the prediction of epitopes covering the maximum population possible. IEDB MHC-II module assigns an IC50 value for each HTL epitope that is inversely related to the binding affinity for receptor MHC-II. An IC50 score less than 50 nM means high binding affinity; less than 500 nM means intermediate binding affinity, whereas an IC50 value less than 5000 nM is related to the low binding affinity of an epitope to the MHC-II receptor [39]. The binding affinity of HTL epitopes predicted by IEDB is inversely related to the percentile rank.

#### 3.2.3. B-Cell Epitope Prediction

The prediction of B-cell epitopes for each RBD of the studied hCoV species was performed using ABCpred (https://webs.iiitd.edu.in/raghava/abcpred/ABC_submission.html, accessed on 15 June 2021) server [40]. This server employs a new method (the kernel method) for the prediction of linear B-cell epitopes. SVM (support vector machine) is a renowned component of kernel techniques that is composed of several algorithms used for pattern analysis. The output performance of ABCpred server (AUC = 0.75) is based on an SVM in combination with amino acid pair antigenicity (AAP) (AUC = 0.7), which is utilized for the prediction of linear B-cell epitopes.

### 3.3. Immunogenic and MHC-I Binding Potential Evaluation

The epitopes, before inclusion in the final vaccine constructs, were evaluated for immunogenic potential (antigenicity and allergenicity) through using two different servers. Similarly, MHC-I binding potentials of the CTL and HTL epitopes were also evaluated.

#### 3.3.1. Antigenicity Profiling

VaxiJen server (http://www.ddg-pharmfac.net/vaxijen/VaxiJen/VaxiJen.html/, accessed on 18 June 2021) [41] was employed to predict the antigenic profile of putative epitopes and final peptide-based vaccine designs. The server’s antigenic capacity prediction is based on the physicochemical features of a specific protein or peptide, and it can be used instead of alignment techniques. It also offers a quite reliable accuracy of 70–89% for antigenicity profiling. Identification of antigenic sequences is based on higher scores (>0.4) than the threshold. On the basis of these selection criteria, only highly antigenic epitopes were short-listed in final vaccine designs.

#### 3.3.2. Allergenicity Profiling

Putative vaccine epitopes and final vaccine constructs were subjected to allergenic profiling using AllerTOP 2.0 server (https://www.ddg-pharmfac.net/AllerTOP/index.html, accessed on 21 June 2021) [42]. AllerTOP 2.0 is an improved version; numerous features were incorporated to improve the performance of the method. The server calculates the allergenic potential of a given amino acid sequence with high specificity by utilizing the k-nearest neighbors algorithm (kNN, k = 1) based on a training set containing 2427 known allergens and nonallergens. The resultant nonallergenic epitopes were further considered in vaccine designs.

#### 3.3.3. MHC-I Binders Profiling

To predict putative binding MHC class I alleles corresponding to short-listed CTL and HTL epitopes in the final vaccine design, IEDB MHC-I binding tool (http://tools.iedb.org/mhci/, accessed on 29 June 2021) [43] was utilized. The tool uses the IEDB server’s specific consensus algorithm for prediction of binding HLA for specific epitopes.

### 3.4. Molecular Docking of CTL and HTL Epitopes and HLAs

The HawkDock server was used for in silico molecular docking of selected T-cell and HTL epitopes with respective HLAs. The server was provided with 3D structures (pdb format) of the HLAs as receptors and the epitopes as ligands. Herein, the 3D models of Human Leukocyte Antigens corresponding to selected T-cell and HTL epitopes were downloaded from the Protein Databank (PDB) [44]. Furthermore, the peptide structures were modeled with an online, available (https://webs.iiitd.edu.in/raghava/pepstrmod/, accessed on 9 July 2021) tool. Additionally, the HawkDock server (http://cadd.zju.edu.cn/hawkdock/, accessed on 14 July 2021) [45] was utilized to predict the binding patterns and calculate the MM/GBSA scores for the best docking complexes.

### 3.5. Vaccine Construction

After short-listing several putative epitopes with qualifying parameters, the process of vaccine construction took place. This included the construction of an mRNA- and a multiepitope-based prophylactic vaccine against the highly pathogenic hCoVs.

#### 3.5.1. mRNA Vaccine Sequence Construction

The formulation of the mRNA vaccine required the addition of several critical components in the open reading frame of the construct. This included 5′ and 3′ UTRs, Kozak sequence, putative epitopes, suitable linkers, and a stop codon. The start codon (AUG) is included in the Kozak sequence [46], whereas the stop codon can be added at the end [47]. Moreover, it is important to select rigid, cleavable, and flexible linkers to allow independent operation of each subunit of the mRNA vaccine and avoid potential interference [48]. In accordance with previous studies [49,50], suitable linkers were chosen on the basis of optimal length and other physical properties. The epitopes (CTL, HTL and B-cell) in the mRNA construct were joined through linkers (AAY, PMGLP, and GGGGS, respectively) [51,52,53]. Additionally, cellular facilitation of antigen presentation of epitopes was ensured with the addition of the tissue plasminogen activator (tPA) secretory signal sequence. This was achieved after acquiring the added tPA protein sequence from UniProtKB by using accession ID: P00750 [54]. Furthermore, to ensure stability of the mRNA-based vaccine design, the addition of elements specific to eukaryotic mRNAs [55,56] was performed. This included the addition of a 5′ cap, poly (A) tail, and 5′ and 3′ untranslated regions. It is vital to choose the optimal length of the poly (A) tail required in efficient translation [57]. Herein, the length of the poly (A) tail was kept between the suggested range of 115–150 nucleotides, in line with previous reports [58]. Moreover, as poly (A) tails operate in tandem with 5′ m7G cap sequences [59], the NCA-7d at the 5′ UTR and S27a + R3U at the 3′ UTR regions were added in the construct to stabilize the proposed mRNA vaccine [60,61].

#### 3.5.2. Multiepitope Vaccine Construction, Structural Modeling, and Validation

All the short-listed epitopes for vaccine designs were subjected to the process of multiepitope-based vaccine construction. A total of 3 CTL and 6 HTL and 6 B-cell epitopes were included in the final prophylactic multiepitope vaccine construct against hCoVs. This was achieved through the use of different linkers (AAY, GPGPG, and KK) to join the epitope sequences in the final constructs. Moreover, adjuvant human beta defensin 2 (HbD-2) was added at the N terminal to improve the stability of the vaccine construct. The finalized MEVC was evaluated for antigenicity and allergenicity status. Furthermore, the 3D structures for the designed MEVC (multiepitope vaccine construct) were modeled through using Robetta server. The structures were also validated through the use of ProSA-web (https://prosa.Servi-ces.came.sbg.acat/prosa.php, accessed on 21 July 2021) [62], PROCHECK (https://servicesn.mbi.ucla.edu/PROCHECK/, accessed on 23 July 2021), and VERIFY3D (https://servicesn.mbi.ucla.edu/Verify3D/, accessed on 29 July 2021). Additionally, the physicochemical properties were evaluated for each of the designed vaccines through Expasy [63].

### 3.6. Molecular Docking Analysis

The designed prophylactic-MEVC-CoV was processed for molecular docking analysis with human TLR4 using the HawkDock server. The server is a multifunctional program that integrates the HawkRank scoring function, the ATTRACT algorithm for docking, and the MM/GBSA free energy decomposition analysis. The molecular mechanical energies and scale solvent methods are combined in the MM/GBSA method [64]. Moreover, the docking analysis of selected T-cell epitopes with corresponding HLAs was also performed. Herein, each HLA 3D structure was retrieved from the Protein Databank (PDB), and HawkDock server (http://cadd.zju.edu.cn/hawkdock/, accessed on 10 August 2021) was utilized to calculate the MM/GBSA score for the best docking poses.

### 3.7. In Silico Cloning and Codon Optimization

The optimized DNA sequence for the final prophylactic-MEVC-CoV design was obtained through the utility of JCat server. The CAI values and GC content for the final vaccine construct sequence were also obtained. The DNA sequence was then inserted into a protein expression plasmid vector (Pet28a+) through using SnapGene. Restriction enzyme (*Xho1* and *EcoR1*) sites were selected to perform in silico cloning and acquire the final plasmid map.

### 3.8. Immune Simulation

In silico immune simulation was carried out to predict the real-life immune system response to the prophylactic-MEVC-CoV design using C-ImmSim server [65]. This simulator employs machine learning (ML) and a position-specific scoring matrix (PSSM) for the prediction of immune system and epitope interactions. The smallest suggested gap between the first and second dose of most vaccines currently in use is 4 weeks [66]. Three injections, each containing 1000 units of the vaccine, were given four weeks apart for our immune simulation. For calculating simulation durations, the C-ImmSim server employs a time-step scale. Each time step on this scale corresponds to 8 h in real life. The total number of time steps for simulation was customized to 1050, and the injection points were set at time steps 1, 84, and 168. The remaining parameters were left at their default values.

## 4. Conclusions

In conclusion, an immunoinformatics approach was utilized to short-list putative immune epitopes for RBDs that were utilized in the final construction of an MEVC for three different hCoVs. Different epitopes, including T-cell, B-cell, and HTL epitopes, were mapped and included in the final vaccine design against the target RBDs. Two different prophylactic vaccines were designed including an mRNA-based vaccine and a proteome-wide multiepitope-based vaccine. Additionally, the short-listed T-cell and HTL epitopes were analyzed through molecular docking with their corresponding HLAs, which demonstrated high binding energies. This step was followed by structural modeling and the evaluation of the potential immunization ability of the constructed MEVC against hCoVs. This study provides new insights into the development of future vaccines against pandemic coronaviruses. The characterization of epitopes and MEVC designs with their evaluated immunogenic potential suggested that experimental processing of these vaccines against hCoVs is needed. However, further experiments may validate the immune reinforcement potential of these putative vaccine candidates.

## Figures and Tables

**Table 1:** The accession ID, domain name, protein name, and position of the selected sequences in the spike RBD of each studied hCoV species.

Accession ID	Domain Name	Protein Name	Organism	Position of RBD in the Spike (GP)
K9N5Q8	BetaCoV S1-CTD	Spike glycoprotein	MERS	381–587
P59594	RBD	Spike glycoprotein	SARS-CoV	306–527
P0DTC2	RBD	Spike glycoprotein	SARS-CoV-2	319–541

**Table 2:** The details of total identified T-cell epitopes and short-listed ones for the vaccine design against each RBD of MERS, SARS, and SARS-CoV-2.

Target Species	Number of Predicted T-Cell Epitopes	Immunogenic T-Cell Epitopes	Position in the RBD
MERS	11	MTEQLQMGF	183–191
SARS-CoV	9	NATKFPSVY	25–33
SARS-CoV-2	9	VGGNYNYLY	127–135

**Table 3:** The details of total identified HTL epitopes and short-listed epitopes for prophylactic vaccine design against each RBD of MERS, SARS, and SARS-CoV-2.

Target Species	Number of Predicted HTL Epitopes	Immunogenic HTL Epitopes	Position in the RBD
MERS	1351	LSMKSDLSVSSAGPI QFNYKQSFSNPTCLI	70–84 86–100
SARS-CoV	1456	NYNYKYRYLRHGKLR SGDVVRFPNITNLCP	130–144 5–19
SARS-CoV-2	1463	TESIVRFPNITNLCP RFASVYAWNRKRISN	5–19 28–42

**Table 4:** The details of total identified B-cell epitopes and short-listed epitopes for the vaccine design against each target RBD sequence of MERS, SARS-CoV, and SARS-CoV-2.

Target Species	Number of Predicted B-Cell Epitopes	Immunogenic B-Cell Epitopes	Position in the RBD
MERS	18	TCLILATVPHNLTTIT TVAMTEQLQMGFGITV	97–112 180–195
SARS-CoV	24	MGCVLAWNTRNIDATS TTTGIGYQPYRVVVLS	112–127 180–195
SARS-CoV-2	23	FSTFKCYGVSPTKLND TGCVIAWNSNNLDSKV	56–71 112–127

**Table 5:** Characterization of CTLs as MHC Class I (MHC-I) and HTLs as MHC Class II (MHC-II) binding epitopes with corresponding HLAs.

Species	Type of Peptide	Peptide Sequence	Position in the RBD	HLA	Binding Energies
MERS	CTL	MTEQLQMGF	183–191	HLA-B*57:01	−27.86 kcal/mol
SARS-CoV	CTL	NATKFPSVY	25–33	HLA-B*35:01	−20.65 kcal/mol
SARS-CoV-2	CTL	VGGNYNYLY	127–135	HLA-A*01:01	−21.87 kcal/mol
MERS	HTL	LSMKSDLSVSSAGPI	70–84	HLA-B*58:01	−37.1 kcal/mol
MERS	HTL	QFNYKQSFSNPTCLI	86–100	HLA-B*15:01	−31.49 kcal/mol
SARS-CoV	HTL	NYNYKYRYLRHGKLR	130–144	HLA-A*24:02	−33.44 kcal/mol
SARS-CoV	HTL	SGDVVRFPNITNLCP	5–19	HLA-A*02:06	−35.7 kcal/mol
SARS-CoV-2	HTL	TESIVRFPNITNLCP	5–19	HLA-B*51:01	−29.69 kcal/mol
SARS-CoV-2	HTL	RFASVYAWNRKRISN	28–42	HLA-A*68:01	−46.27 kcal/mol

## Data Availability

Not applicable.

## Supplementary Materials

The following supporting information can be downloaded at: https://www.mdpi.com/article/10.3390/molecules27072375/s1. Figure S1—Showing docking complexes of HTL epitopes for each CoV specie with respective HLAs; Figure S2—Showing interaction patterns of individual HTL epitopes with respective HLAs; Table S1—Showing details of binding scores and identified interaction patterns between the different immune epitopes and respective HLAs; Table S2—Showing details free energy calculations for the different CTL and HTL epitopes and respective HLAs; Table S3—Showing physiochemical properties including of the proposed MEVC.
