# Supplementary material for: Potential Immunogenic Activity of Computationally Designed mRNA- and Peptide-Based Prophylactic Vaccines against MERS, SARS-CoV, and SARS-CoV-2: A Reverse Vaccinology Approach
Source: Molecules. 2022 Apr 6;27(7):2375. doi: 10.3390/molecules27072375 (PMC9000378; doi:10.3390/molecules27072375)
Supplement: Supplementary file 1 [file molecules-27-02375-s001.zip › molecules-1609300-supplementary.pdf]

molecules

MDPI

Supplementary Information

Potential immunogenic activity of computationally designed Spike-RBD epitopes based prophylactic vaccine against MERS, SARS-CoV and SARS-CoV-2, A reverse vaccinology approach

Taimoor Khan[1], Abbas Khan[1], Jawad Khaliq Ansari[2], Muzammil Hasan Najmi[2], Dong-Qing Wei[1,3,4],Khalid Muhammad[5*], Yasir Waheed[2*]

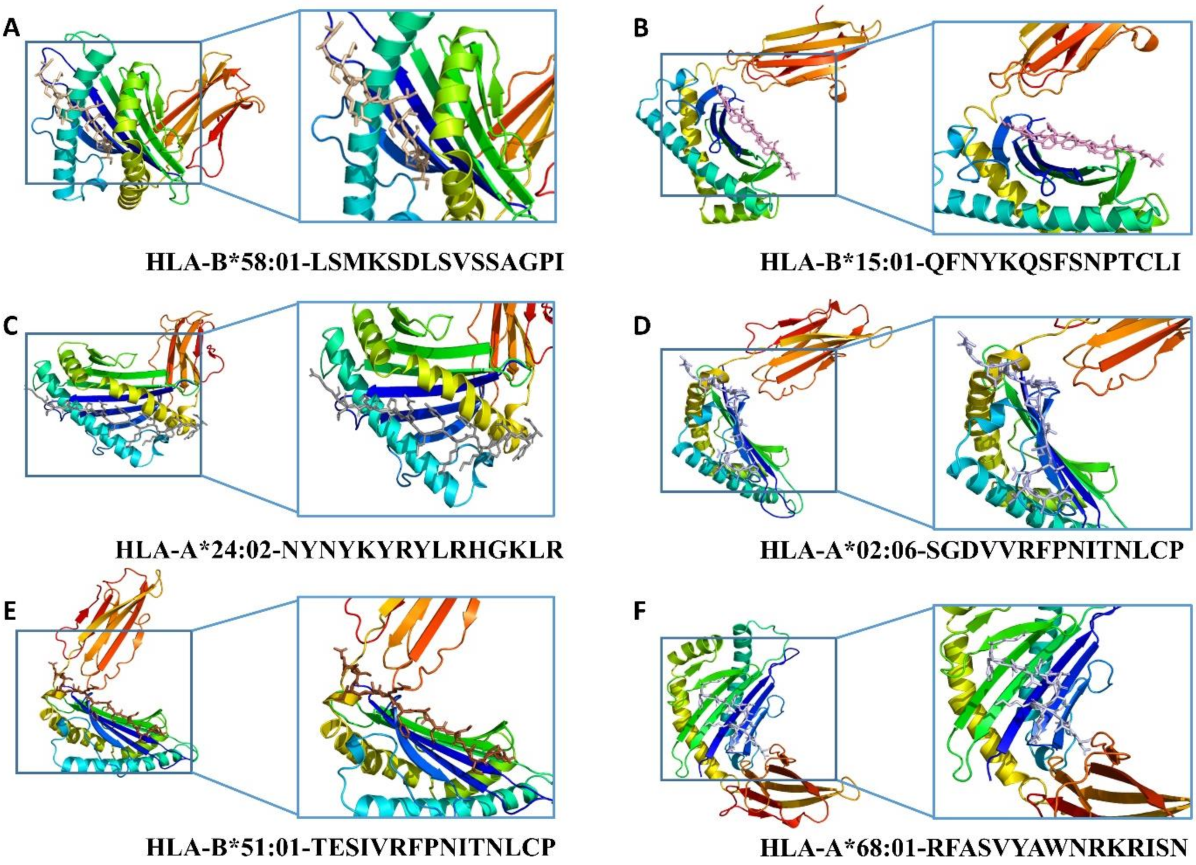

Figure S1. Showing docking complexes of HTL epitopes for each CoV specie with respective HLAs.

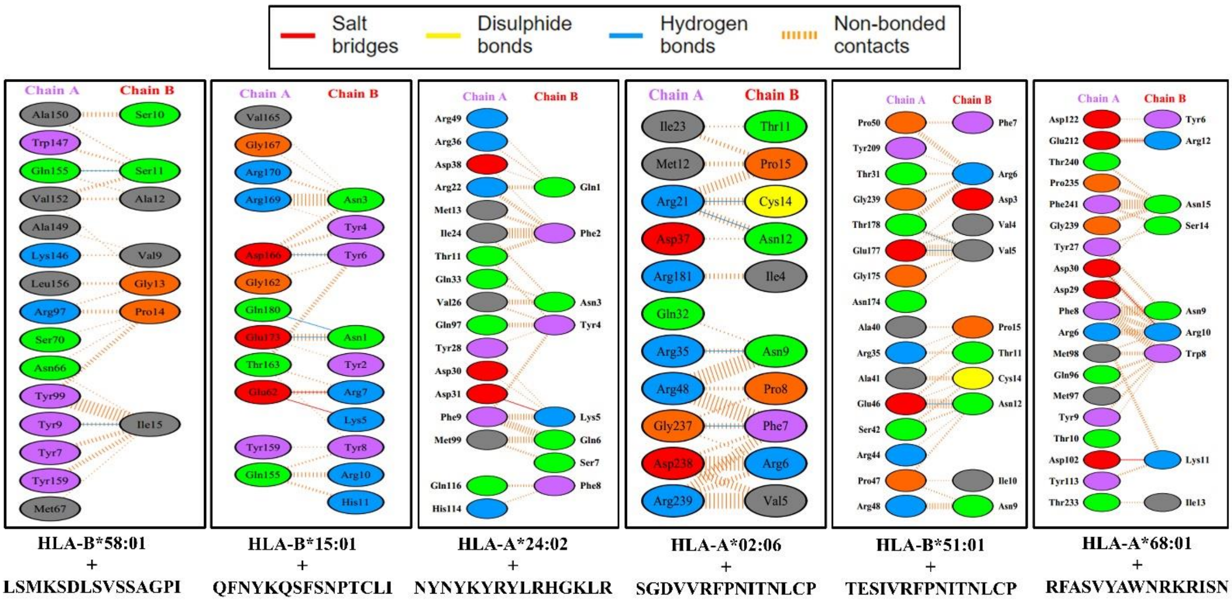

Figure S2. Showing interaction patterns of individual HTL epitopes with respective HLAs.

Table S1. Showing details of binding scores and identified interaction patterns between the different immune epitopes and respective HLAs.

Epitope + HLA complex	Epitope position in the RBD domain	hCOV specie	Docking Score	Binding free energy	Number of Hydrogen Bonds	Number of Salt Bridges	Number of non-bonded contacts
**MTEQLQMGF + HLA-B*57:01**	183-191	MERS-CoV	-2244.26	-27.86 kcal/mol	5	-	92
**NATKFPSVY + HLA-B*35:01**	25-33	SARS-CoV	-2426.57	-20.65kcal/mol	3	-	140
**VGGNYNYLY + HLA-A*01:01**	127-135	SARS-CoV-2	-2945.07	-21.87kcal/mol	5	-	142
**LSMKSDLSVSSAGPI + HLA-B*58:01**	70-84	MERS-CoV	-2334.26	-37.1kcal/mol	2	-	105
**QFNYKQSFSNPTCLI + HLA-B*15:01**	86-100	MERS-CoV	-2785.36	-31.49 kcal/mol	3	2	132
**NYNYKYRYLRHGKLR + HLA-A*24:02**	130-144	SARS-CoV	-3279.04	-33.44 kcal/mol	-	1	141
**SGDVVRFPNITNLCP + HLA-A*02:06**	5-19	SARS-CoV	-2602.58	-35.7 kcal/mol	4	-	141
**TESIVRFPNITNLCP + HLA-B*51:01**	5-19	SARS-CoV-2	-2728.46	-29.69 kcal/mol	3	-	118
**RFASVYAWNRKRISN + HLA-A*68:01**	28-42	SARS-CoV-2	-3669.67	-46.27 kcal/mol	-	3	189

Table S2. Showing details free energy calculations for the different CTL and HTL epitopes and respective HLAs.

Complex name	Epitope position in the RBD Domain	VDW	ELE	GB	SA	TOTAL
**MTEQLQMGF + HLA-B*57:01**	183-191	-48.52	-59.59	87.51	-7.26	-27.86
**NATKFPSVY + HLA-B*35:01**	25-33	-62.66	-255.21	305.77	-8.55	-20.65
**VGGNYNYLY + HLA-A*01:01**	127-135	-52.34	-12.09	49.95	-7.39	-21.87
**LSMKSDLSVSSAGPI + HLA-B*58:01**	70-84	-40.5	-36.04	46.18	-6.75	-37.1
**QFNYKQSFSNPTCLI + HLA-B*15:01**	86-100	-60.45	-161.38	198.28	-7.94	-31.49
**NYNYKYRYLRHGKLR + HLA-A*24:02**	130-144	-65.9	-723.97	765.14	-8.72	-33.44
**SGDVVRFPNITNLCP + HLA-A*02:06**	5-19	-60.84	-134.77	168.83	-8.92	-35.7
**TESIVRFPNITNLCP + HLA-B*51:01**	5-19	-54.92	-127.72	160.74	-7.79	-29.69
**RFASVYAWNRKRISN + HLA-A*68:01**	28-42	-74.1	-602.08	640.31	-10.41	-46.27

Table S3. Showing physiochemical properties including of the proposed MEVC.

Complex name	Epitope position in the RBD Domain	VDW	ELE	GB	SA	TOTAL
**MTEQLQMGF + HLA-B*57:01**	183-191	-48.52	-59.59	87.51	-7.26	-27.86
**NATKFPSVY + HLA-B*35:01**	25-33	-62.66	-255.21	305.77	-8.55	-20.65
**VGGNYNYLY + HLA-A*01:01**	127-135	-52.34	-12.09	49.95	-7.39	-21.87
**LSMKSDLSVSSAGPI + HLA-B*58:01**	70-84	-40.5	-36.04	46.18	-6.75	-37.1
**QFNYKQSFSNPTCLI + HLA-B*15:01**	86-100	-60.45	-161.38	198.28	-7.94	-31.49
**NYNYKYRYLRHGKLR + HLA-A*24:02**	130-144	-65.9	-723.97	765.14	-8.72	-33.44
**SGDVVRFPNITNLCP + HLA-A*02:06**	5-19	-60.84	-134.77	168.83	-8.92	-35.7
**TESIVRFPNITNLCP + HLA-B*51:01**	5-19	-54.92	-127.72	160.74	-7.79	-29.69
**RFASVYAWNRKRISN + HLA-A*68:01**	28-42	-74.1	-602.08	640.31	-10.41	-46.27
